# Indirect Force Control of a Cable-Driven Parallel Robot: Tension Estimation using Artificial Neural Network trained by Force Sensor Measurements

**DOI:** 10.3390/s19112520

**Published:** 2019-06-01

**Authors:** Jinlong Piao, Eui-Sun Kim, Hongseok Choi, Chang-Bae Moon, Eunpyo Choi, Jong-Oh Park, Chang-Sei Kim

**Affiliations:** 1 School of Mechanical Engineering, Chonnam National University, Gwangju 61186, Korea; piaojinlong622@gmail.com (J.P.); hs.choi@jnu.ac.kr (H.C.); cbmoon@jnu.ac.kr (C.-B.M.); eunpyochoi@jnu.ac.kr (E.C.); 2 Medical Microrobot Center, Robot Research Initiative, Chonnam National University, Cheomdangwagi-ro, Buk-gu, Gwangju 61011, Korea; eskim9357@jnu.ac.kr

**Keywords:** cable-driven parallel robot, pulley friction, artificial neural network, force control, cable tension estimation, cable force sensor

## Abstract

In a cable-driven parallel robot (CDPR), force sensors are utilized at each winch motor to measure the cable tension in order to obtain the force distribution at the robot end-effector. However, because of the effects of friction in the pulleys and the unmodeled cable properties of the robot, the measured cable tensions are often inaccurate, which causes force-control difficulties. To overcome this issue, this paper presents an artificial neural network (ANN)-based indirect end-effector force-estimation method, and its application to CDPR force control. The pulley friction and other unmodeled effects are considered as black-box uncertainties, and the tension at the end-effector is estimated by compensating for these uncertainties using an ANN that is developed using the training datasets from CDPR experiments. The estimated cable tensions at the end-effector are used to design a P-controller to track the desired force. The performance of the proposed ANN model is verified through comparisons with the forces measured directly at the end-effector. Furthermore, cable force control is implemented based on the compensated tensions to evaluate the performance of the CDPR in wrench space. The experimental results show that the proposed friction-compensation method is suitable for application in CDPRs to control the cable force.

## 1. Introduction

A cable-driven parallel robot (CDPR) is a special type of parallel robot, which is actuated by elastic cables instead of rigid links. A CDPR consists of a fixed frame, robot end-effector, winch-motors, and cable pulleys. The robot end-effector motion is controlled by the length and tension of each cable, and each cable is driven by each respective winch system. The tension generated by the winch motor is transmitted to the end-effector through connected elastic cables guided by pulleys. Generally, the length and tension of each cable are measured using an encoder and load cell at the winch side. This type of measurement does not cause cable and/or sensor interferences to the end-effector’s movement. The lightweight elastic cable actuator provides the CDPR with the advantages of high payload capability, large workspace, and fast dynamics. As a result, the CDPR has many possible applications such as in large telescopes [1], 3D printing [2], and high-speed manipulation [3].

However, the CDPR has practical control problems induced by kinematic redundancy, nonlinear elastic-cable behavior, and pulley friction. As the cables can only pull and not push the end-effector, the number of cables must be higher than the necessary number of end-effector’s degrees-of-freedom (DOF). Based on the number of cables (*m*) and number of DOFs (*n*), CDPRs can be classified into: Under-constrained type (*m* < *n*) and fully-constrained type (*m* ≥ *n* + 1) [4]. Amongst these, the fully-constrained CDPR is essential for implementing full 6-DOF robotic motion. However, the fully-constrained CDPR has infinite tension distribution for any given spatial posture of the end-effector, and its kinematic structure matrix depends strongly on the pose of the end-effector in the parallel robotic mechanism. This brings forth another important issue as to how all the actuating cables can be maintained at the proper tension levels by considering the redundant kinematic constraints, to avoid sagging of cables or overloading; that is, eventually, for the CDPR force control.

Several force-distribution algorithms [5,6,7] were previously utilized for CDPR motion control and cable-tension control [8,9,10]. However, even with these force-distribution algorithms, the CDPR force control was challenging, in practice. One main reason was the difficulty of measuring the direct force distributions at the end-effectors, because of the friction between the cables and pulleys [11]. Friction modeling and compensation methods have been widely studied for robot systems, and some methods were proposed for cable–pulley friction models in the CDPR. With the assumption of no-slip at the cable and pulley interface, Choi et al. modeled the pulley bearing friction based on the Dahl friction, to estimate cable tensions during slow motion [12]. Kraus et al. implemented pulley friction compensation using the Coulomb and Dahl friction model. They also analyzed the influence of friction on the wrench measurement and pointed out that pulley friction influenced the wrench hysteresis significantly [11]. Peng et al. used a multibody dynamic approach to model the friction of the cable–pulley system. The model considered the friction of the sliding joint, using the arbitrary Lagrangian–Eulerian formulation [13]. In addition, for a diverse range of mechanical systems requiring accurate positioning and force control, many research has addressed the pulley friction effect on the cable–pulley structured mechanisms [14,15,16,17].

The CDPR friction between the cable and pulley is influenced by the pulley bearing friction, sliding friction, and friction among other mechanical parts. In this context, it can be stated that most of the traditional-model-based friction estimations, used in the previous research, represented only part of the friction phenomena. Moreover, the previous methods experienced difficulties in identifying the model parameters, especially for dynamic models because of the nonlinearity and long-term variations of properties due to abrasion [18]. Moreover, in addition to pulley friction, the cable elongation and its nonlinear properties also caused tension discrepancies for the cable connection from each winch to the end-effector. Therefore, a more complicated model for accurate tension estimation is necessary.

In this paper, we propose an artificial neural network (ANN)-based indirect end-effector force-estimation method, and design a proportional CDPR force-control algorithm that can compensate for the inaccurate measurements of the CDPR end-effector force and achieve indirect force control for CDPR. We consider the pulley friction and unmodeled cable effects as a black-box uncertainty. The estimated cable tension of the end-effector compensates for this uncertainty by using an ANN model that is derived from the training datasets of the CDPR experiments. In order to implement an efficient ANN model, we obtain the parameters affecting the friction in a CDPR based on the traditional friction model. Then, the ANN model is trained by spatial motion experiments conducted for the given trajectories that cover the desired motion ranges of the end-effector. The MATLAB ANN toolbox is used to design an ANN estimator, which is implemented in real-time using PLC structured-text programming. The effectiveness of the proposed ANN model is evaluated based on the cable force control achieved in the CDPR system.

It is well known that ANNs have the capability to approximate nonlinear functions through learning processes [19,20,21,22], and they were previously applied for friction compensation, as follows. Huang et al. used two neural network (NN) approximators to design an intelligent controller for compensating the effect of nonlinear friction in a 1-DOF mechanical system [23]. Guo et al. proposed an NN structure with additional jump approximation activation functions to model the complex and discontinuous friction dynamics for a si*x*-axis articulated robot tracking control [24]. Liu et al. used an NN-based friction compensation method for approximating the residual values during free motion [25]. Several other friction compensation techniques using ANNs could be found in the previous research [26,27,28]. Nevertheless, to the best of the authors’ knowledge, our study is the first ANN application to use the cable force control of a CDPR by using an ANN-based CDPR end-effector’s cable tension estimator for estimating the cable–pulley friction and compensating for cable uncertainties.

The outline of this paper is as follows. Section 2 introduces the laboratory-scale CDPR, which is employed in this study, and explains the robot kinematics and dynamics. Section 3 describes the friction effects in the cable–pulley compartments and provides an ANN-model-based friction-compensation method. In Section 4, the force and position control algorithms based on ANN are explained. Section 5 shows the experimental results of the ANN-based cable force control in a Mini CDPR. Finally, we conclude this paper in Section 6.

## 2. CDPR System

### 2.1. MINI CDPR

**Figure 1:**
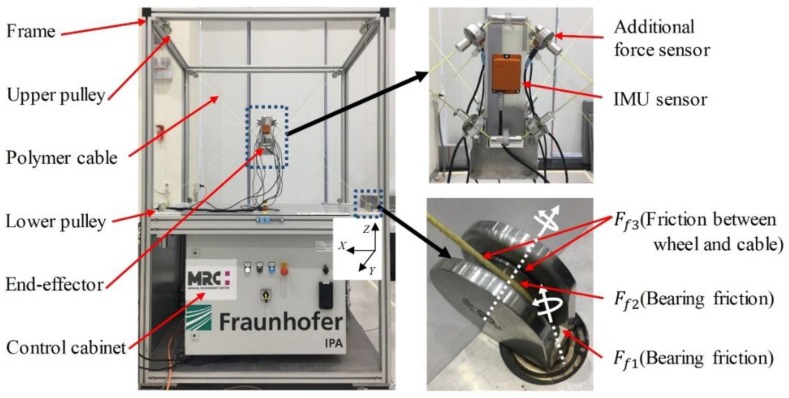
Photograph of the MINI cable robot and its cable connection components.

The lab-scale Mini CDPR employed in this work is shown in Figure 1. It is a type of fully-constrained 6-DOF CDPR, which is actuated by eight polyethylene Dyneema^®^ cables (LIROS D-Pro 01505-0200, 2 mm). The robot uses industrial servo drives and controllers as hardware, and TwinCAT3 is used as the real-time control software [29]. The size of the fixed frame is 1.10 m × 0.80 m × 0.95 m, and the mass of the end-effector is 1.42 kg. Force sensors (Micro Load Cell - CZL635 up to 50 N) for cable force measurements are integrated with each guiding pulley at the winch motors at the bottom of the CDPR frame. This mechanism can prevent the interferences from additional sensors, to the end-effector movement. However, the cable force measurements are always affected by the friction between the winch and the end-effector.

In this study, in order to obtain the gold-standard force distribution of the end-effector, eight additional force sensors (UMM-K10 up to 100 N) are connected directly to the end-effector for reference measurements. It should be pointed out that the additional force sensors are installed only for the friction and uncertainty identification and ANN training.

### 2.2. Force Sensor Calibration

**Figure 2:**
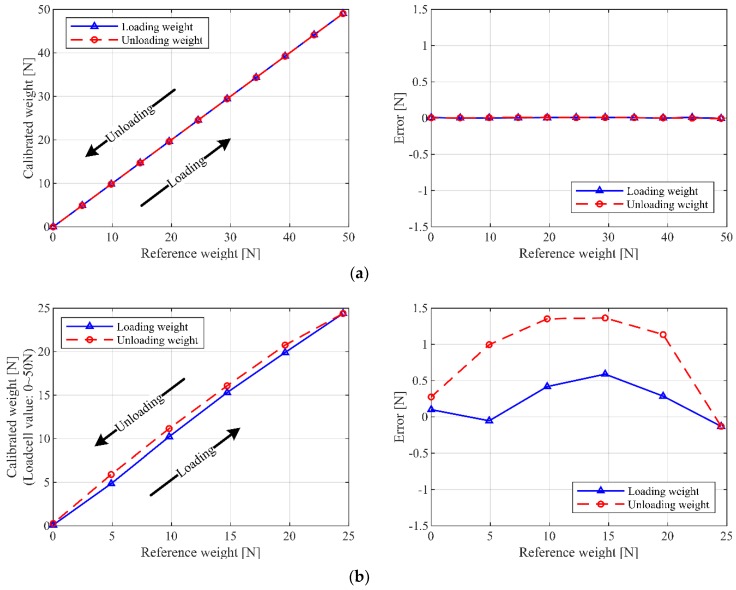
Force sensor calibration results: (**a**) Calibration without pulley; (**b**) calibration with pulley.

**Figure 3:**
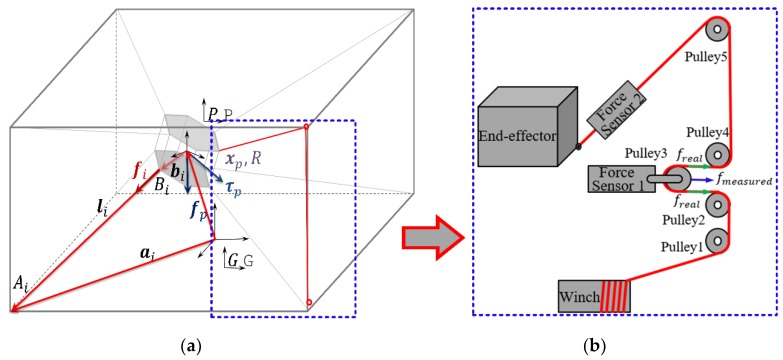
Description of the robot geometry: (**a**) Kinematic and dynamic notation of cable-driven parallel robot (CDPR); (**b**) cable pulley system.

Each force sensor must be calibrated, to avoid unnecessary issues arising from the manufacturing variations and/or influence of the operating environment. In order to achieve the best possible accuracy, we perform two types of calibration approaches: Calibration without pulley and calibration with pulley. In the former approach, the force sensor is calibrated directly by attaching known weights; in the latter, the force sensor is combined with a cable pulley system and is calibrated by attaching known weights. The performances of the calibrations are compared with the experimental results, as shown in Figure 2. According to the wiring mechanism of the pulley in Figure 3b, the measured value from force sensor 1 is half of the real cable tension. In the case of calibration with a pulley, hysteresis is observed between the loading and unloading processes. Furthermore, the maximum error is approximately 1.5 N, which is 6% of the actual cable tension capacity. On the other hand, for the calibration without pulley, very good accuracy is observed, as the system is not influenced by pulley friction. Hence, all the force sensors are calibrated without using pulleys to obtain pure tension values. 

### 2.3. Kinematics and Dynamics

Figure 3a shows a schematic of the fully-constrained CDPR, where the index i denotes the cable number. The geometric parameter $A_{i}$ is a cable-attaching point on the base frame and $B_{i}$ is a cable-attaching point on the end-effector. $a_{i}$ and $b_{i}$ are two constant vectors of the base coordinate {G} and end-effector coordinate {P}, respectively. The inverse kinematics are used to describe the joint variables from the given end-effector posture [30]. Hence, based on the closure vector loop, the cable length vector $l_{i}$ can be described as

(1) $$l_{i}=a_{i}-x_{p}-Rb_{i}$$

where $x_{p}$ and R describe the end-effector position and orientation, respectively. 

In Figure 3a, $f_{i}$ denotes the cable force vector along the i-th cable, while the external force $f_{p}$ and torque $\tau_{p}$ act on the end-effector. The force and torque equilibrium at the end-effector yields

(2) $$\overset{m}{\underset{i=1}{\sum }}f_{i}+f_{p}=0$$

(3) $$\overset{m}{\underset{i=1}{\sum }}\left(Rb_{i}\times f_{i}\right)+\tau_{p}=0$$

where $f_{i}=f_{i}u_{i}$ with the obtained unit vector being $u_{i}=l_{i}/∥l_{i}∥$. From Equations (2) and (3), the equilibrium equation can be written in the matrix form as follows.

(4) $$\underset{A^{T}}{\underbrace{\left(\begin{array}{ccc} u_{i} & \cdots & u_{m}\\ Rb_{i}\times u_{i} & \cdots & Rb_{m}\times u_{m} \end{array}\right)}}\left(\begin{array}{c} f_{1}\\ \vdots \\ f_{m} \end{array}\right)+\underset{w}{\underbrace{\left(\begin{array}{c} f_{p}\\ \tau_{p} \end{array}\right)}}=0$$

which can be expressed in a more compact form as below:

(5) $$A^{T}\left(x_{p},R\right)f+w=0$$

where w is an external wrench force applied at the end-effector. Here, the transpose of the Jacobian matrix $A^{T}$ is called the structure matrix. The standard dynamic equation of the CDPR, assuming that all the cables are tensioned, can be obtained from Equation (5), in the global coordinate of the robot system, as follows [31].

(6) $$M\left(x\right)\ddot{x}+C\left(x,\dot{x}\right)\dot{x}+G+F_{f}\left(x,\dot{x}\right)=-A^{T}f$$

where M(x) is the inertia matrix of the robot, $C\left(x,\dot{x}\right)$ is the vector of Coriolis and centripetal terms, G is the vector of gravity terms, and $F_{f}\left(x,\dot{x}\right)$ is the friction term in the system.

## 3. ANN-Based Cable Tension Estimation

An ANN is a generalized model of the biological nervous system, which is based on the brain function, to obtain the knowledge. It has the ability to learn effectively from data, and compute nonlinear problems. The main advantage of the ANN model is that it can be implemented easily without complicated mathematical modeling and parameter identification. As the traditional methods often experience difficulty in modeling complicated nonlinear behavior, intelligent ANN-based friction compensation is designed in this study.

### 3.1. Friction Model Revisited

In CDPR, accurate measurement of the force distribution of the end-effector is essential to achieve CDPR force control. However, the measured tensions are influenced by the friction between the force sensor and end-effector and cable effects along the wiring. In the Mini CDPR, five pulleys are used to guide cables to the end-effector and the force sensor is equipped at pulley3, as illustrated in Figure 3b. The friction between the force sensor 1 and end-effector can be divided into two parts, pulley bearing friction and sliding friction. In Figure 1, $F_{f3}$ is the sliding friction between the cable and wheel. $F_{f1}$ and $F_{f2}$ describe two different pulley bearing frictions. $F_{f1}$ is caused by the rotation of the pulley and $F_{f2}$ is due to the rotation of the wheel. 

The basic model to describe the above friction is a combination of the Coulomb friction and linear viscous friction models. It is mathematically given by:

(7) $$F_{f}\left(v\right)=\mu F_{n}sign\left(v\right)+\sigma_{v}v$$

where $F_{n}$ is the normal force. μ and $\sigma_{v}$ are the Coulomb friction coefficient and viscous friction coefficient, respectively. This model exhibits easy implementation, but leads to poor performance, especially in the case of friction at low velocities. 

The Dahl model is a simple model to simulate ball-bearing friction. This model is a function of the displacement x, which makes it possible to estimate the hysteresis behavior of the friction [11]:

(8) $$\frac{dF_{f}\left(x\right)}{dx}=\sigma_{0}{\left|1-\frac{F_{f}}{F_{c}}sign\left(\dot{x}\right)\right|}^{n}sign\left(1-\frac{F_{f}}{F_{c}}sign\left(\dot{x}\right)\right)$$

where $\sigma_{0}$ is the stiffness parameter at an equilibrium point, $F_{c}$. is the Coulomb friction, and n is a material-dependent parameter. The Dahl model can produce a smooth transition around zero velocity, but fails to describe the stiction and Stribeck effect. 

Despite these traditional models, many complex models have been proposed to describe the nonlinear behavior of friction, such as the Karnopp model, Leuven model, and seven parameters model [32]. However, these models present complexities during mathematical modeling and identification of parameters. Instead, we utilized the traditional models, which is revisited in this section, to obtain the key parameters while designing the ANN models used in this study.

### 3.2. Designing and Training the ANN

**Figure 4:**
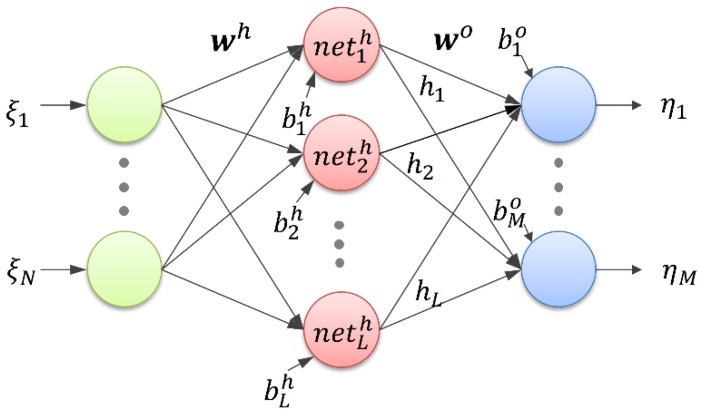
Proposed structure of the artificial neural network.

By considering a characteristic of the CDPR that has the capability in large translational motion but limited rotational motion, we only consider the translational motion in this paper. In order to design and train the proposed neural network, a number of inputs and known outputs are necessary. The input values should be eight measured tensions near the pulleys (***PUL-tension***) and the output values are eight measured reference tensions near the end-effector (***EE-tension***). However, one type of input value is not sufficient to output the desired tension because of the hysteresis and nonlinear behavior of friction during dynamic motion. From Equation (7) and (8) of the traditional pulley friction models, we can see that $F_{f1}$, $F_{f2},$ and $F_{f3}$ are related to the cable length, cable velocity, and pulley wrapping angle, respectively. Therefore, these variables are included in the ANN model. The bearing friction $F_{f1}$ is dependent on the position and velocity of the end-effector. The cable length, cable velocity, and pulley wrapping angle are also dependent on the position and velocity of the end-effector. Hence, we add the position and velocity of the end-effector as additional inputs. The design of the ANN structure is described in Figure 4. In total, 14 inputs and 8 outputs are incorporated in the ANN to compensate for the cable tension discrepancy between the measurements at the winch side and end-effector; the inputs are $\left[\xi_{1},\;\cdots ,\xi_{14}\right]=\left[x_{dx},\;x_{dy},\;x_{dz},\;{\dot{x}}_{dx},\;{\dot{x}}_{dy},\;{\dot{x}}_{dz},\;f_{PUL1},\;\cdots ,f_{PUL8}\right]$ and the outputs are $\left[\eta_{1},\;\cdots ,\eta_{8}\right]=\left[f_{NN1},\;\cdots ,f_{NN8}\right]$. The output of the designed ANN is computed as follows:

(9) $$\eta_{k}=\overset{L}{\underset{j=1}{\sum }}w_{kj}^{o}\;\sigma \left(net_{j}^{h}\right)+b_{k}^{o}$$

where the sigmoid activation functions are $\sigma \left(net_{j}^{h}\right)=1/\left\{1+e^{-\sigma \left(net_{j}^{h}\right)}\right\}$ and $net_{j}^{h}=\;\overset{N}{\underset{i=1}{\sum }}w_{ji}^{h}\;\xi_{i}+b_{j}^{h}$. 

**Figure 5:**
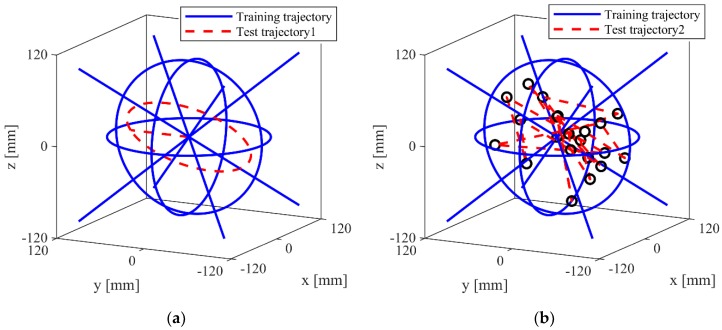
Training trajectories (blue line) and test trajectories (red dot line) for artificial neural network (ANN) model evaluation: (**a**) Test trajectory 1: A three-dimensional circular path and one straight line in the y-direction; (**b**) test trajectory 2: Line paths that connect 20 arbitrary points where the test trajectories are identical.

To obtain an acceptable range of training data, we operate the robot with a predefined trajectory. The training trajectory is a translation motion in space, which includes four straight paths along a diagonal line of cubic space and three circular paths (xy plane, xz plane, and yz plane), as shown in Figure 5. 

We use the MATLAB Toolbox (MathWorks, USA) to create an ANN. In the present study, the Bayesian regularization method is used for friction compensation because this algorithm can produce good generalization for difficult, small, or noisy datasets. It is a two-layer feed-forward network, which consists of sigmoid hidden neurons and linear output neurons to fit the input and output.

### 3.3. Performance Evaluation of ANN

**Figure 6:**
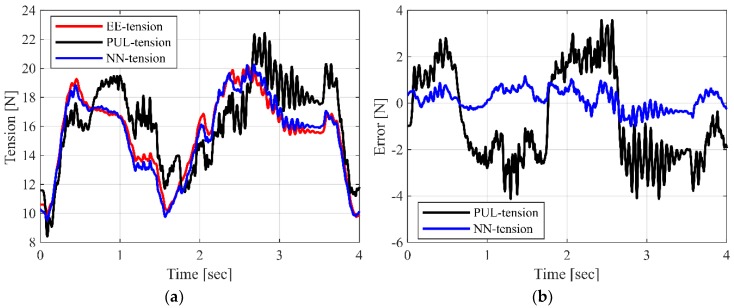
Comparison results of cable tension during movement along test trajectory 1: (**a**) Tension comparison among the measured ***EE-tension***, ***PUL-tension,*** and compensated ***NN-tension***; (**b**) tension error comparison: ***PUL-tension*** versus ***EE-tension*** and ***NN-tension*** versus ***EE-tension***.

Once the ANN has been trained for satisfactory performance, it is tested for performance validation in compensating for the friction in real-time. For the validation, we connect additional force sensors to each cable at the end-effector side, which measure the actual end-effector cable tension (***EE-tension***), as shown in Figure 1. To evaluate the proposed ANN model, the tension measured by the pulley-integrated force sensor (***PUL-tension***) at the winch side and the end-effector tension estimated via the ANN (***NN-tension***) are compared with the tension measured at the end-effector (***EE-tension***) while operating the robot along two kinds of test trajectories, which are different from the training trajectory. The first trajectory is composed of a three-dimensional circular path and one straight line in the y-direction, as shown in Figure 5a. This trajectory is used to evaluate the whole performance of ANN in this paper. The second trajectory is line paths, which connect 20 arbitrary points in the training workspace, as shown in Figure 5b, which for the evaluation of the general case. The root mean square errors (RMSE) between the measured ***PUL-tension*** and measured ***EE-tension*** and between the compensated ***NN-tension*** and measured ***EE-tension,*** for the eight cable tensions, are listed in Table 1 and Table 2. In Figure 6, the ***PUL-tension***, ***NN-tension***, and ***EE-tension*** for a single cable (cable #2, test trajectory 1), and their errors, are plotted. It is observed that the ***PUL-tension*** and ***EE-tension*** are mismatched because of the uncertainty and pulley friction. In cable #2, the maximum error between the measured tension, ***PUL-tension,*** and realistic distributed tension, ***EE-tension,*** is approximately ±4 N. Furthermore, in the eight cables, the RMSE in test trajectory 1 is approximately 2 N and the RMSE in test trajectory 2 is approximately 1.5 N. On the other hand, the tension values, which are compensated by the neural network (***NN-tension***), are very close to the actual end-effector cable tension, ***EE-tension***. The mean value of the RMSE between ***NN-tension*** and ***EE-tension*** is approximately 0.5 N. Hence, we can confirm that the trained ANN force-discrepancy-compensation model can estimate the cable force distribution of the end-effector successfully.

## 4. End-Effector Force Control

**Figure 7:**
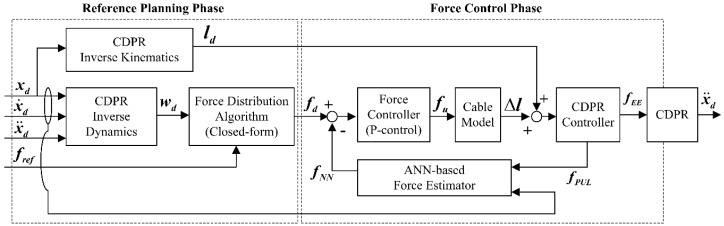
Block diagram of the proposed force control algorithm using the ANN force estimator.

The CDPR force controller based on the ANN end-effector cable-tension estimation is designed for end-effector force tracking, with respect to the predefined cable tensions, as the end-effector moves along the planned trajectory. A block diagram of the proposed force-control algorithm utilizing the ANN cable tension estimation is shown in Figure 7. The position of the CDPR is determined by an open-loop position control mechanism and the force of the end-effector is determined by a closed-loop cable tension control mechanism. The open-loop position control is derived by using the inverse kinematics of the CDPR to obtain the desired cable length $l_{d}$ for the given desired position $x_{d}$, as expressed in Equation (1).

In the fully-constrained CDPR, infinite force-distribution solutions exist because of the non-square structure matrix. Hence, it is necessary to find a suitable range of force distributions, to improve the performance of the CDPR, e.g., to prevent sagging or minimize tension efforts. In this work, we utilized a closed-form solution to calculate the required cable forces [8], to find continuous force distributions quickly, in real time. This algorithm uses the Moore–Penrose matrix inverse $A^{+T}$ to obtain the least-square optimal solution. For the given wrench, $w_{d}$, the desired cable force can be generated as

(10) $$f_{d}=f_{ref}-A^{+T}\left(w_{d}+A^{T}f_{ref}\right)$$

where $f_{ref}$ is the reference cable force. It helps the cable force distribution to be bound in the specified range. However, this formula has a limitation on finding whole feasible solutions. It might fail to provide feasible solutions when close to the border of the wrench-feasible workspace. In this case, an improved force distribution algorithm can be applied to find feasible solutions [33]. In this study, the operating workspace is initially designed by a calculation from the feasible closed form solution, then the feasible solutions are guaranteed at any posture inside the workspace.

While moving, the cable tensions are measured by the force sensors equipped at each winch pulley. However, as the measured ***PUL-tension***, $f_{PUL}$, is different from the actual end-effector force because of uncertainty and cable–pulley friction, we utilize the estimated ***NN-tension***, $f_{NN}$, from the designed ANN as a force-feedback signal. A simple P-controller is implemented to control the tension error between the desired tension and ***NN-tension***. In Figure 7, $f_{u}$ is each cable’s tension control input, which is defined as $f_{u}=k_{p}\left(f_{d}-f_{NN}\right)$. As stated earlier, $f_{NN}$ is obtained to compensate for the tension discrepancy between the measured tension (***PUL-tension***) and actual distributed tension (***EE-tension***) at the end-effector. If we assume that $f_{NN}$ can estimate the actual end-effector tension, then the wrench input can be expressed as follows

(11) $$-A^{T}f≅-A^{T}\left(f_{pos}+k_{p}\left(f_{d}-f_{NN}\right)\right)+C\left(x,\dot{x}\right)\dot{x}+F_{f}\left(x,\dot{x}\right)$$

where $f_{pos}$ is a position-control input to the CDPR. By utilizing Equation (11), the CDPR force-control model can be simplified as follows

(12) $$M\left(x\right)\ddot{x}=-A^{T}\left(f_{pos}+k_{p}\left(f_{d}-f_{NN}\right)\right)-G$$

where $f_{pos}$ is a servo portion of the controller in CDPR, and we consider it in the Cartesian space as $-A^{T}f_{pos}=k_{sv}\dot{e}+k_{sp}e$. Then, the servo portion is obtained by $f_{pos}=k_{mv}{\dot{e}}_{m}+k_{mp}e_{m}$, so we can derive the relationship between Cartesian space and Joint space as $k_{sv}\dot{e}+k_{sp}e=-A^{T}\left(k_{mv}{\dot{e}}_{m}+k_{mp}e_{m}\right)$, where $k_{mp}$ and $k_{mv}$ are proportional and derivative (PD) parameters in joint space, respectively. Thus, we can obtain the following error dynamics for the CDPR system:

(13) $$\begin{array}{cc} & M\left(x\right)\ddot{e}+k_{sv}\dot{e}+k_{sp}e\\ = & A^{T}k_{p}\left(f_{ref}-A^{+T}\left(w_{d}+A^{T}f_{ref}\right)-f_{NN}\right)+M\left(x\right){\ddot{x}}_{d}+G\\ = & 0 \end{array}$$

where $A^{T}A^{+T}=I$ and $w_{d}=M\left(x\right){\ddot{x}}_{d}+G$.

Let us define a Lyapunov candidate as $V=1/2\left(e^{2}+{\dot{e}}^{2}\right),$ which is positive definite. The derivative of the Lyapunov function can be negative definite, according to the LaSalle’s invariance principle as follows.

(14) $$\begin{array}{cc} \dot{V} & =e\dot{e}+\dot{e}\ddot{e}\\ & =e\dot{e}+\dot{e}\left\{M^{-1}\left(-k_{sv}\dot{e}-k_{sp}e\right)\right\}\\ & =-\dot{e}M^{-1}k_{sv}\dot{e}\leq 0. \end{array}$$

Hence, the asymptotic stability of the designed controller can be verified. 

Finally, the input tension to the CDPR is conveyed to the cable length input through the winch motor. We use the linear stiffness model to transfer the cable force to the cable length, as follows

(15) $$\Delta l_{f}=\;\frac{l}{AE}f_{u}$$

where E is the elastic modulus, A is the actual cross-sectional area, and l is the cable length between each winch and the attaching point of the end-effector. 

## 5. Experimental Results

**Figure 8:**
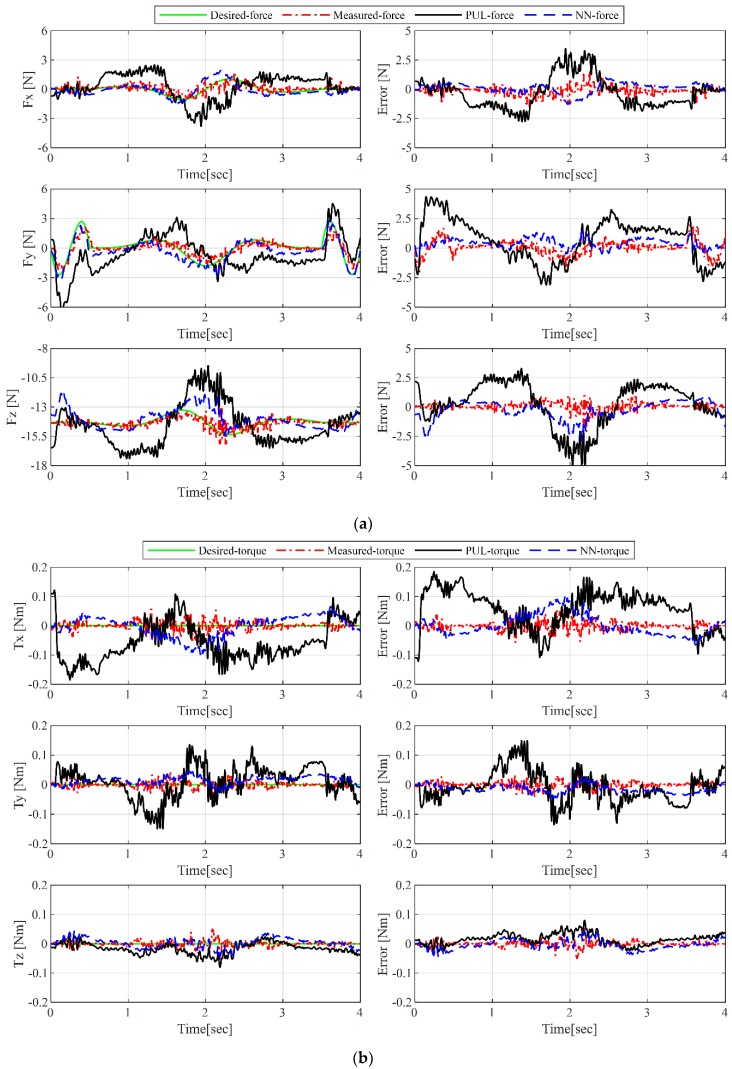
Results of cable force control in terms of (**a**) force and force errors; and (**b**) torque and torque errors.

The CDPR force control is performed using the proposed control scheme, as shown in Figure 7, in the test trajectory 1. We use original factory setting values of the control gain $k_{mp}=diag\left[0.015\right]$ and $k_{mv}=diag\left[0.125\right]$ (drive model: EL7201-BECKHOFF). In the case of cable force control, the P gain ($k_{p}=diag\left[0.70\right]$) is tuned manually and selected by the sensitivity analysis as listed in Table 3. In order to evaluate the performance of cable tension control in the wrench space, in addition to the additional force sensors along the cable at the end-effector side, an inertia measuring unit (IMU) sensor (MTI-300, Netherlands) is assembled at the end-effector, which can measure the actual end-effector force during the experiments. In Figure 8, the force and torque applied to the end-effector are expressed in four ways, where the desired force and torque are calculated with the given accelerations, based on Equation (6). Simultaneously, the measured-force and torque are obtained from the IMU sensor measurements. In the results, ***PUL-force*** and ***PUL-torque*** are the calculated force and torque from the measured ***PUL-tension***, and ***NN-force*** and ***NN-torque*** are obtained from the estimated ***NN-tension*** via ANN. Furthermore, the RMSEs between ***Desired-wrench*** and ***Measured-wrench***, ***Desired-wrench*** and ***NN-wrench***, and ***Desired-wrench*** and ***PUL-wrench*** are listed in Table 4.

The experimental results show that the actual end-effector force obtained by both the IMU sensor and ANN algorithm follow the desired force references within acceptable ranges. However, the ***PUL-force*** shows poor performance in the wrench space, compared to both the IMU sensor measurements and desired force. This is because of the tension discrepancy between the realistic distributed tension (***EE-tension***) and measured ***PUL-tension*** from the winch side. This result emphasizes the fact that the use of uncompensated tension measurements to control the robot can lead to undesirable performances. If the test trajectory involves only translation motion, the desired orientation motion and torque value will be zero. In the case of applications such as pick and place, which only require translation motion and force, unwanted orientation motion and torque may influence the performance of the robot. Hence, the orientation and torque accuracy are analyzed as well, from these experiment results. We also verify the performance of the proposed ANN algorithm; the torque measurements from the IMU sensor and the ***NN-torque*** from the estimated ***NN-tension*** are very close, with RMSE less than 0.03 Nm, which can be considered as sensor noise. 

**Figure 9:**
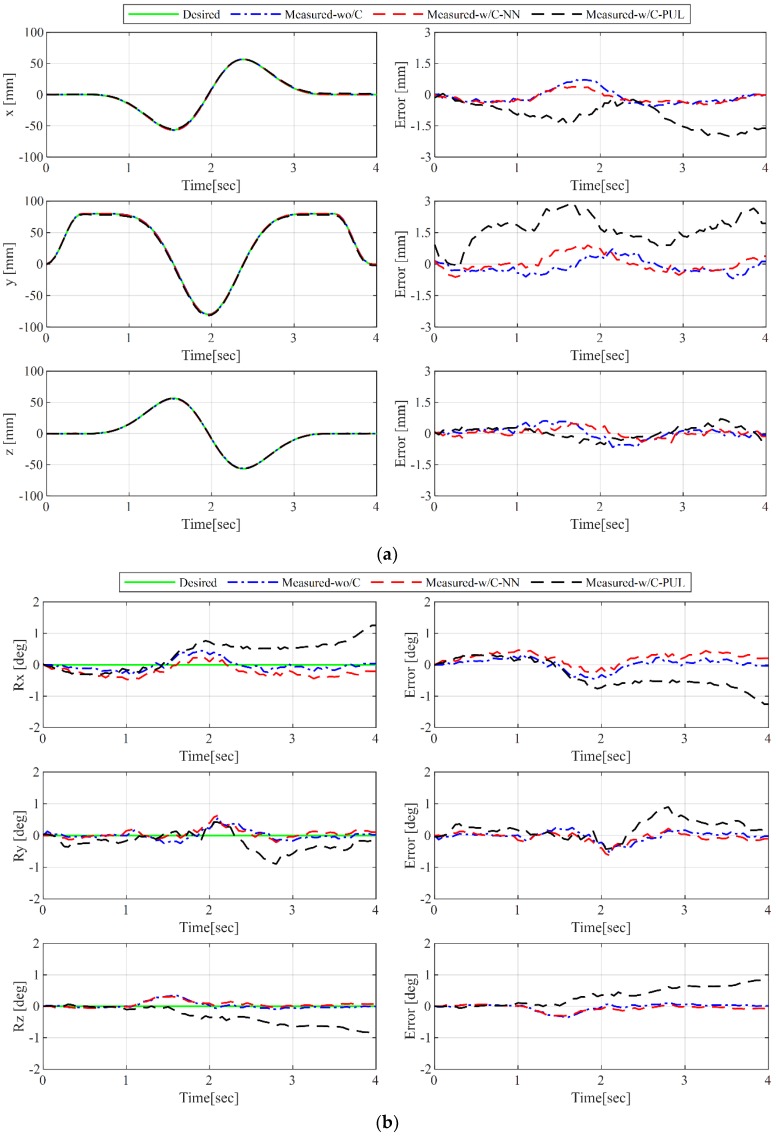
Comparison of position and orientation accuracy, with and without cable force control: (**a**) Positions and position errors; (**b**) orientations and orientation errors. Measured-wo/c: Measured position and orientation without force control, measured-w/C-NN: Measured position and orientation with force control based on the estimated ***NN-tension***, measured-w/C-PUL: Measured position and orientation with force control based on the measured ***PUL-tension***.

To evaluate the position trajectory tracking performance, a precise 6-DOF displacement measurement sensor, OTS (optical track sensor, Model: Polaris Spectra from NDI^®^ with RMS of 0.3 mm at 60 Hz) is utilized. In Figure 9, the position and orientation accuracy are compared for the cases of without cable force control, with ***NN-tension***-based force control, and with ***PUL-tension***-based force control. In the case of without force control and with ***NN-tension***-based force control, the position errors are less than 1 mm and orientation errors are less than 0.6°. However, the position and orientation errors of ***PUL-tension***-based force control are quite large. These results show that inaccurate cable tension distribution can be affecting the balance of the end-effector, which lead to the inaccurate performance of the robot.

**Figure 10:**
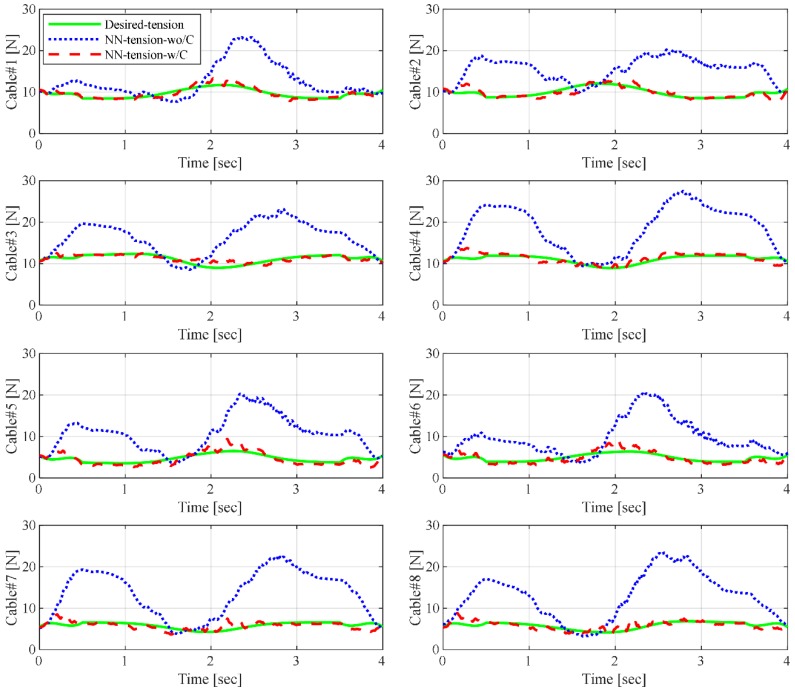
Comparison of cable tension in cable-driven parallel robot (CDPR) with and without cable force control. NN-tension-wo/C: Compensated neural network tension without force control during motion; NN-tension-w/C: Compensated neural network tension with force control during the motion.

Figure 10 shows the CDPR cable tension comparison during force-control experiments: ***NN-tension*** without force control and ***NN-tension*** with force control. When cable force control is not applied, the cable tension changes over a wide range. The cable tension in cable #4 is greater than 25 N, which is out of the accurate range of the force sensor measurement. However, when we apply the proposed cable tension control, the actual cable tension follows the desired tension. This is a strong proof that the proposed ANN-based end-effector cable-tension estimation and force-control scheme can control the individual cable tensions within certain bounds of desired tension ranges, which can prevent sagging or overloading during movement. 

## 6. Conclusions

In this paper, we presented an ANN-based end-effector force estimation method to compensate for the uncertainties including cable–pulley friction and nonlinear cable behavior that created tension discrepancies between the tensions measured at the winch side and end-effector. The proposed ANN model designed by position and velocity measurements could estimate the force distribution of the end-effector, accurately, from the available tension measurements at the winch pulley side. The performance of the designed ANN model was verified by the experimental results for ANN model validation and CDPR end-effector force control. The results showed that the estimated end-effector force from the designed ANN model could overcome the cable tension discrepancies caused by uncertainties in the force transmission to the end-effector from the winch motor. Moreover, the measured force distributions in the time domain were very close to the actual values and could be applied to the CDPR force controller as cable tension feedback. Finally, the proposed method could control the cable tensions within the desired range, which could prevent sagging and minimize the required tension energy. In the future, the developed method can be implemented in grand CDPR systems designed for handling heavy loads. An advanced control algorithm for a force and position hybrid controller will be developed to enhance the application of CDPR in the industry.

## Figures and Tables

**Table 1:** Root mean square error (RMSE) between measured ***EE-tension*** (end-effector tension measurements), measured ***PUL-tension*** (winch-motor-side tension measurements), and estimated ***NN-tension*** (end-effector tension estimated by ANN) in test trajectory 1.

[N]	$f_{1}$	$f_{2}$	$f_{3}$	$f_{4}$	$f_{5}$	$f_{6}$	$f_{7}$	$f_{8}$	Mean
***EE-tension*** vs. ***PUL-tension***	1.81	2.11	2.02	2.77	1.89	1.69	1.81	1.82	1.99
***EE-tension*** vs. ***NN-tension***	0.82	0.46	0.42	0.65	0.34	0.44	0.64	0.30	0.51

**Table 2:** Root mean square error (RMSE) between measured ***EE-tension*** (end-effector tension measurements), measured ***PUL-tension*** (winch-motor-side tension measurements), and estimated ***NN-tension*** (end-effector tension estimated by ANN) in test trajectory 2.

[N]	$f_{1}$	$f_{2}$	$f_{3}$	$f_{4}$	$f_{5}$	$f_{6}$	$f_{7}$	$f_{8}$	Mean
***EE-tension*** vs. ***PUL-tension***	1.34	1.54	1.30	1.41	1.63	1.57	1.31	1.50	1.45
***EE-tension*** vs. ***NN-tension***	0.52	0.51	0.65	0.48	0.38	0.67	0.36	0.73	0.54

**Table 3:** Force error sensitivity analysis (all cables have the same gain) with respect to the gain variation.

*k_p_*	0.55	0.60	0.65	0.70	0.75	0.85	0.90
***RMSE* [*N*]**	0.489	0.466	0.465	0.437	0.451	0.479	0.527

**Table 4:** Root mean square error (RMSE) between *Desired-wrench*, *Measured-wrench*, *NN-wrench*, and *PUL-wrench*.

	$F_{x}\left[N\right]$	$F_{y}\left[N\right]$	$F_{z}\left[N\right]$	$T_{x}\left[Nm\right]$	$T_{y}\left[Nm\right]$	$T_{z}\left[Nm\right]$
***Desired-wrench*** vs. ***Measured-wrench***	0.42	0.61	0.37	0.01	0.01	0.01
***Desired-wrench*** vs. ***NN-wrench***	0.44	0.56	0.80	0.03	0.02	0.02
***Desired -wrench*** vs. ***PUL-wrench***	1.36	1.90	1.87	0.09	0.05	0.03
